# F183. FUSIFORM GYRUS ABNORMALITIES RELATED TO VERBAL INTELLIGENCE AND NEGATIVE SYMPTOMS IN FIRST-EPISODE PSYCHOSIS

**DOI:** 10.1093/schbul/sby017.714

**Published:** 2018-04-01

**Authors:** Sang-Hyuk Lee, Jeong Hoon Kim, Sra Jung, Arira Lee

**Affiliations:** 1 CHA Bundang Medical Center, CHA University; 2 Bundang Jesaeng Hospital

## Abstract

**Background:**

Researches have shown that verbal intelligence of first-episode psychosis (FEP) can be deteriorated. Only a few studies have investigated about neural correlates of verbal intelligence in FEP. Fusiform gyrus is often referred to the “visual word form area (VWFA)”, also known to be systematically activated by reading. The object of this study is to explore the relationship between the volume of language processing regions and the verbal IQ (VIQ) in FEP.

**Methods:**

102 patients with FEP and 54 HC were enrolled this study. All subjects were right-handed. All patients were interviewed and diagnosed with the diagnostic criteria in Structured Clinical Interview for DSM-5 and examined by means of MRI at 3 Tesla at base line. Schizophrenia patients were measured their IQ through Korean-Wechsler Adult Intelligence Scale (K-WAIS). Positive and Negative Syndrome Scale (PANSS) were administered at baseline and 8 weeks after antipsychotics administration for patients. We used the FreeSurfer software package for estimating the volume of language processing area including pars triangularis, pars opercularis, insula, Heschl’s gyrus, Planum temporale and fusiform gyrus of left dominant hemisphere.

**Results:**

Compared to the HCs, first-episode psychosis patients showed volume reductions in fusiform gyrus (p=0.000) and pars opercularis (p=0.006) of left hemisphere among language related regions after Bonferoni correction. We found that only the volume of fusiform gyrus of left hemisphere showed significant positive correlation with VIQ (r=0.30, p=0.026) and also showed positive correlation with its subscales (vocabulary subscale (p=0.042), arithmetic WAIS subscale (p=0.012), similarities subscale (p=0.034)). In addition, the volume of fusiform gyrus of left hemisphere were significantly negative correlated with the score of PANSS Negative subscale at 8 weeks (p=0.016) after antipsychotics administration.

**Discussion:**

These findings suggest that the fusiform gyrus can be related to the verbal intelligence in first-episode psychosis patients and it may be associated with the severity of negative symptoms after treatment.

